# Policing the legume-Rhizobium symbiosis: a critical test of partner choice

**DOI:** 10.1038/s41598-017-01634-2

**Published:** 2017-05-03

**Authors:** Annet Westhoek, Elsa Field, Finn Rehling, Geraldine Mulley, Isabel Webb, Philip S. Poole, Lindsay A. Turnbull

**Affiliations:** 1 Department of Plant Sciences, University of Oxford, Oxford, OX1 3RB UK; 2 Systems Biology Doctoral Training Centre, University of Oxford, Oxford, OX1 3RQ UK; 3 Department of Ecology, Philipps-University Marburg, Marburg, D-35043 Germany; 4 School of Biological Sciences, University of Reading, Reading, RG6 6AJ UK

## Abstract

In legume-*Rhizobium* symbioses, specialised soil bacteria fix atmospheric nitrogen in return for carbon. However, ineffective strains can arise, making discrimination essential. Discrimination can occur via partner choice, where legumes prevent ineffective strains from entering, or via sanctioning, where plants provide fewer resources. Several studies have inferred that legumes exercise partner choice, but the rhizobia compared were not otherwise isogenic. To test when and how plants discriminate ineffective strains we developed sets of fixing and non-fixing strains that differed only in the expression of *nifH* – essential for nitrogen fixation – and could be visualised using marker genes. We show that the plant is unable to select against the non-fixing strain at the point of entry, but that non-fixing nodules are sanctioned. We also used the technique to characterise mixed nodules (containing both a fixing and a non-fixing strain), whose frequency could be predicted using a simple diffusion model. We discuss that sanctioning is likely to evolve in preference to partner choice in any symbiosis where partner quality cannot be adequately assessed until goods or services are actively exchanged.

## Introduction

Across the globe primary productivity is nitrogen limited^1^. This limitation has been overcome for plants in the family Fabaceae (commonly known as legumes) through a mutualistic association with nitrogen-fixing bacteria collectively called rhizobia^2^. The nitrogen provided through this symbiosis makes legumes rich in protein and important crops in human diets^3^. But, as ineffective strains will inevitably arise through mutation, there is the potential for the relationship to break down. Ineffective strains are known to be common, at least in some situations, which for agricultural legumes means poor yields and reduced nutritional quality^4, 5^.

Theory predicts that ineffective strains could be successful within legume – *Rhizobium* symbioses for two reasons^6, 7^. First, rhizobia are not transmitted directly from parent plant to offspring. Instead, plants acquire rhizobia from the soil through an intricate signalling process in which bacteria enter specialized root nodules, where they fix nitrogen in return for plant-derived carbon^8^. This horizontal transmission means that rhizobial fitness is not perfectly aligned with the fitness of the host plant^7^. Second, although each nodule is usually occupied by the clonal descendants of a single *Rhizobium* ^9, 10^, a plant is usually infected by multiple rhizobial strains^11, 12^. Thus, a non-fixing strain can potentially thrive by taking plant resources while leaving the costly process of nitrogen fixation to others^7, 13^. To prevent losing resources to ineffective rhizobial strains that provide little or no nitrogen, legumes have two options: partner choice or sanctions^7^.

Partner choice is usually defined as any mechanism that allows detection of suitable partners *before* a mutualistic relationship is established^7, 13–15^, while sanctioning is a mechanism to discriminate against low-quality partners once the relationship is underway^6, 7, 13–16^ (although confusingly ‘partner choice’ has also been used to describe a broader concept which includes sanctioning)^13, 17, 18^. Partner choice might seem to be the more attractive option as resources are not wasted setting up a relationship that is doomed to fail. But crucially, effective partner choice requires accurate assessment of the quality of partners in advance^13^. This is likely to be problematical for any symbiosis in which key traits are not manifested prior to the relationship being established. For example, in the legume-*Rhizobium* symbiosis, nitrogen fixation does not begin until the bacteria have entered the roots and nodule formation is sufficiently advanced for rhizobia to have differentiated into nitrogen-fixing bacteroids^8^. Once nodules are established they can be sanctioned, if they prove to be ineffective, by cutting off their supply of carbon, oxygen or other nutrients and this has been demonstrated empirically using argon gas to force nodules to fix less nitrogen^19–21^.

Despite the empirical support for sanctioning and its apparent advantages, there are nevertheless several studies that claim evidence for partner choice^7, 14, 15^. However, the interpretation of these studies is problematic because the tested strains are rarely isogenic – meaning that strains differ in several traits, and not just in how much nitrogen they provide. Most importantly, strains are likely to differ in their competitiveness in colonizing plant roots and forming nodules. A range of traits affects competitiveness: examples include motility^22^, production of antibiotics^23^ and the secretion of proteins and polysaccharides involved in biofilm formation and root attachment^24^. Such differences in competitiveness explain why poorly-fixing strains can also end up occupying a higher proportion of nodules – a problem that is often encountered when developing effective strains for use in agricultural settings^4, 25, 26^. Thus comparing the nodulation success of naturally occurring strains is difficult to interpret as a test of partner choice.

To test whether plants can directly assess the effectiveness of potential rhizobia prior to nodulation we created a non-fixing mutant from a fixing strain and compared their success in colonising pea plant nodules. There are several key genes involved in nitrogen fixation^8^, any of which could undergo a mutation that would render the gene non-functional and hence transform the fixing into a non-fixing strain. We chose the *nifH* gene in *Rhizobium leguminosarum* bv. viciae (*Rlv*) 3841, and created a non-fixing mutant that was otherwise identical to its fixing parent strain. We then assessed when and how the plant discriminated between the two strains. To identify strains, they were marked with *gusA* or *celB* marker genes, rendering strains magenta or blue (respectively) following the application of a simple post-harvest staining protocol. Insertion of marker genes solves a secondary problem as it is usually extremely time-consuming to identify different strains using antibiotic markers and this limits the number of nodules that can be assessed.

One possible complication is that a non-fixing strain can potentially thrive via mixed nodules (where two different bacteria have entered and colonised). If a non-fixing strain can take advantage of mixed nodules to increase its fitness at the expense of the fixing strain, then this would provide a route by which non-fixing strains could increase in frequency. Currently little is known about the frequency at which mixed nodules occur, and the relative fitness of strains within mixed nodules. The staining protocol rendered mixed nodules easily visible, so we assessed the frequency of mixed nodules under different inoculation densities. We discuss mixed nodules, partner choice and sanctioning in the context of the evolutionary stability of the legume-*Rhizobium* symbiosis.

## Results

**Figure 1:**
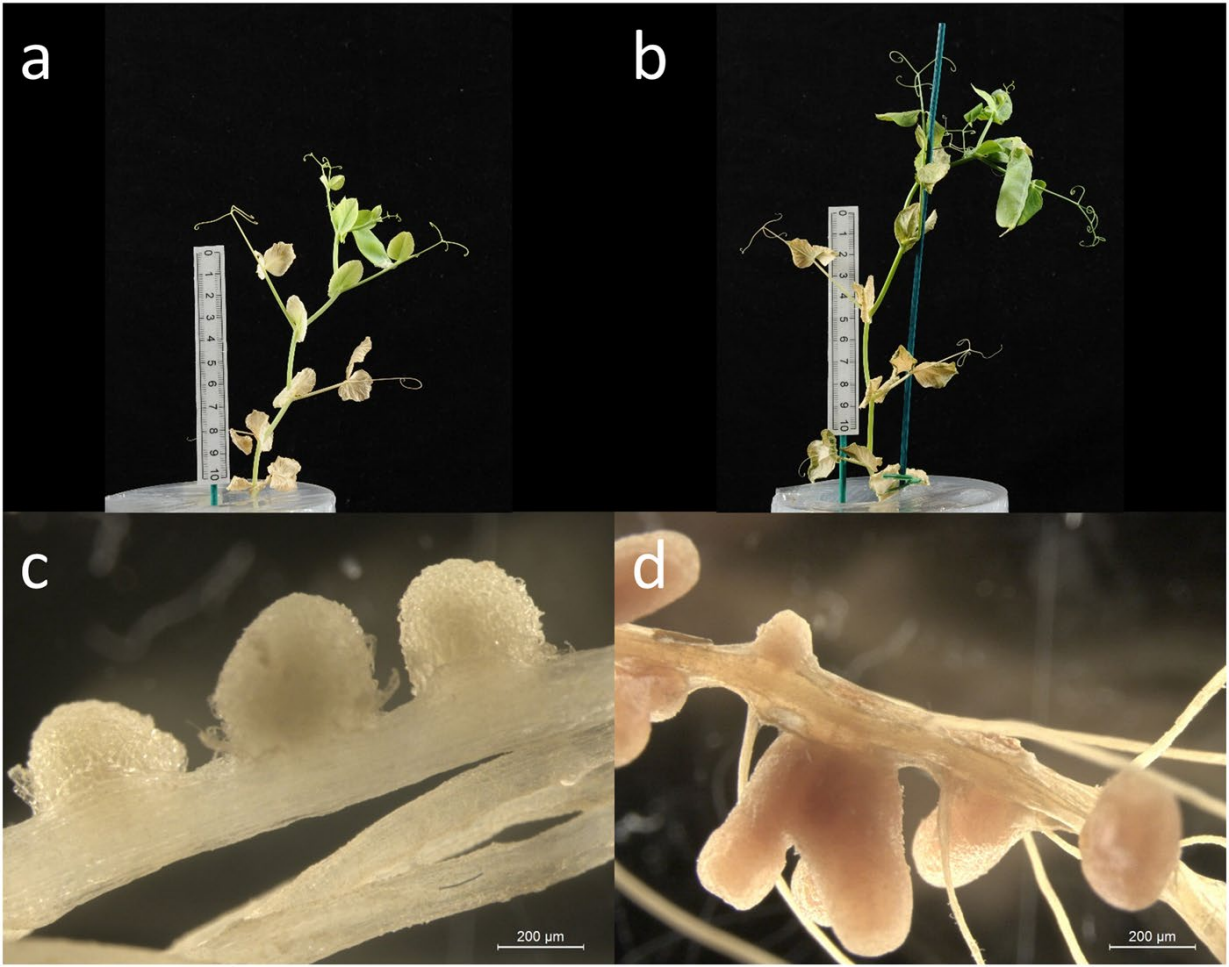
Comparison of the non-fixing strain RU3940, (**a**,**c**) and the otherwise isogenic fixing strain Rlv3841 (**b**,**d**). After five weeks of growth, plants inoculated with the non-fixing strain (**a**) had reduced biomass and produced fewer peas than those inoculated with the fixing strain (**b**). The non-fixing strain formed white (**c**), rather than pink (**d**), nodules, indicating a lack of leghaemoglobin. Scale bars indicate 200 μm.

In single inoculations, the non-fixing mutant strain reduced shoot mass and formed small white nodules, while the fixing parent strain formed larger pink nodules (indicating the presence of leghaemoglobin) (Fig. 1).

### Partner choice

**Figure 2:**
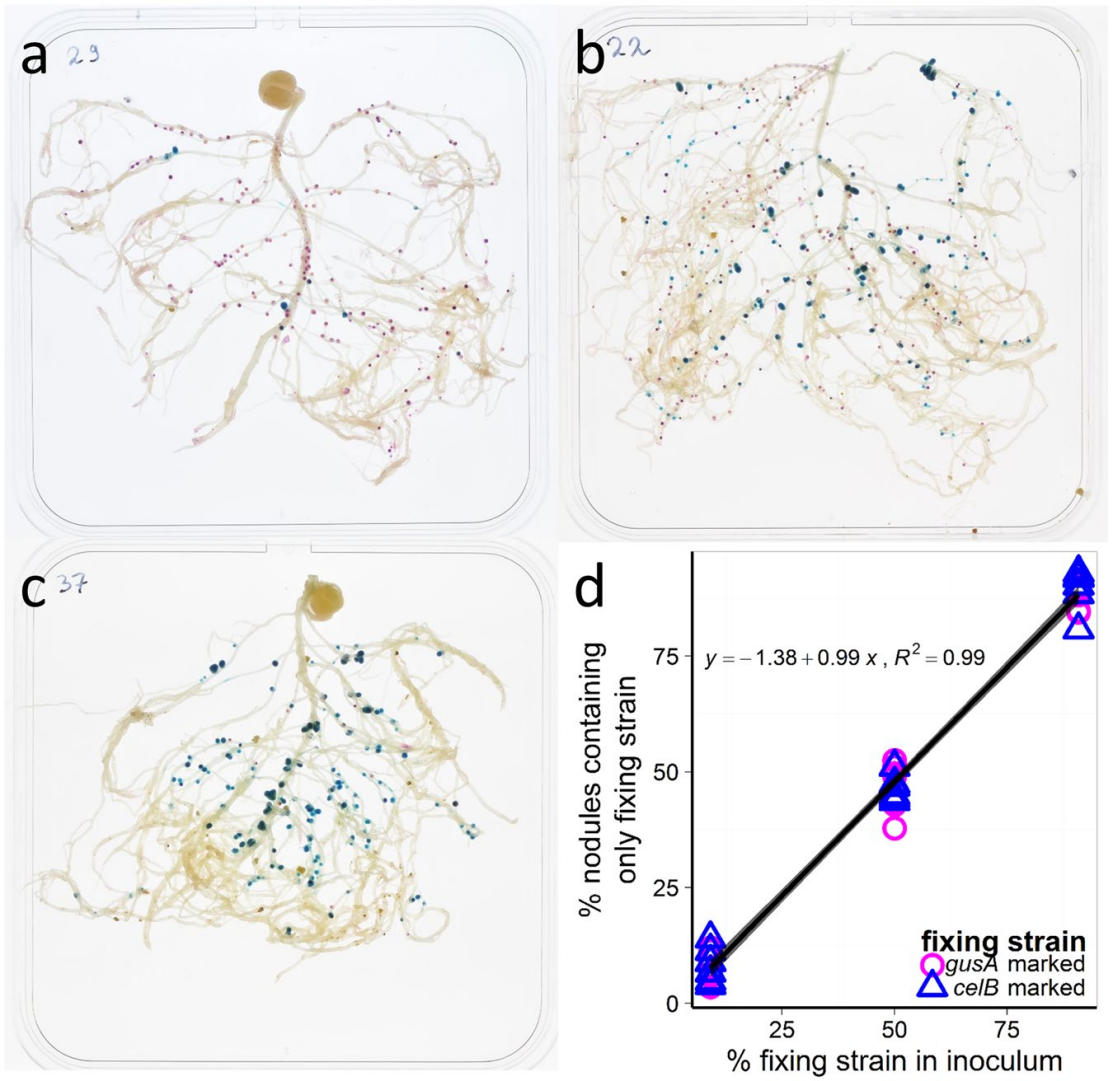
We found no evidence of partner choice. (**a**–**c**) show pea root systems with stained nodules following inoculation with different ratios of a fixing *celB* (blue) marked strain (3841 *celB*) and a non-fixing *gusA* (magenta) marked mutant strain (OPS0365): (**a**) 1:10 (fixing:non-fixing); (**b**) 1:1 (fixing:non-fixing); (**c**) 10:1 (fixing:non-fixing). (**d**) The percentage of nodules containing only the fixing strain exactly reflected the percentage of the fixing strain in the inoculum. The slope of the regression was 0.99 ± 0.02, 95% CI [0.95, 1.02] and this is not significantly different from one (*t*_39_ = −0.835, *p* = 0.41).

To test for partner choice, we varied the ratio of the fixing: non-fixing strains in the inoculum (1:10, 1:1 and 10:1) and counted the number of fixing and non-fixing nodules after three weeks. In the absence of partner choice, we expect the percentage of nodules containing the fixing strain to reflect the percentage in the inoculum. In other words, we expect a 1:1 relationship between the percentage of fixing nodules and the percentage of the fixing strain in the inoculum (a slope of 1.0 through the origin). We found that the percentage of nodules containing only the fixing strain exactly reflected the percentage of the fixing strain in the inoculum (Fig. 2). The slope of the regression line was 0.99 ± 0.02 (95% CI [0.95, 1.02]) and this is not significantly different from 1.0 (*t*_39_ = −0.835, *p* = 0.41). The intercept was not significantly different from zero (*t*_39_ = −1.338, *p* = 0.189). Whether the fixing strain was marked with *gusA* or *celB* had no impact on the proportion of fixing nodules formed (*t*_37_ = −0.177, *p* = 0.861) or on the interaction with the inoculum ratio (*t*_37_ = −0.189, *p* = 0.851). Thus, pea plants do not discriminate between fixing and non-fixing strains prior to nodule formation and are therefore unable to exercise partner choice against the mutant strain.

### Nodule size

**Figure 3:**
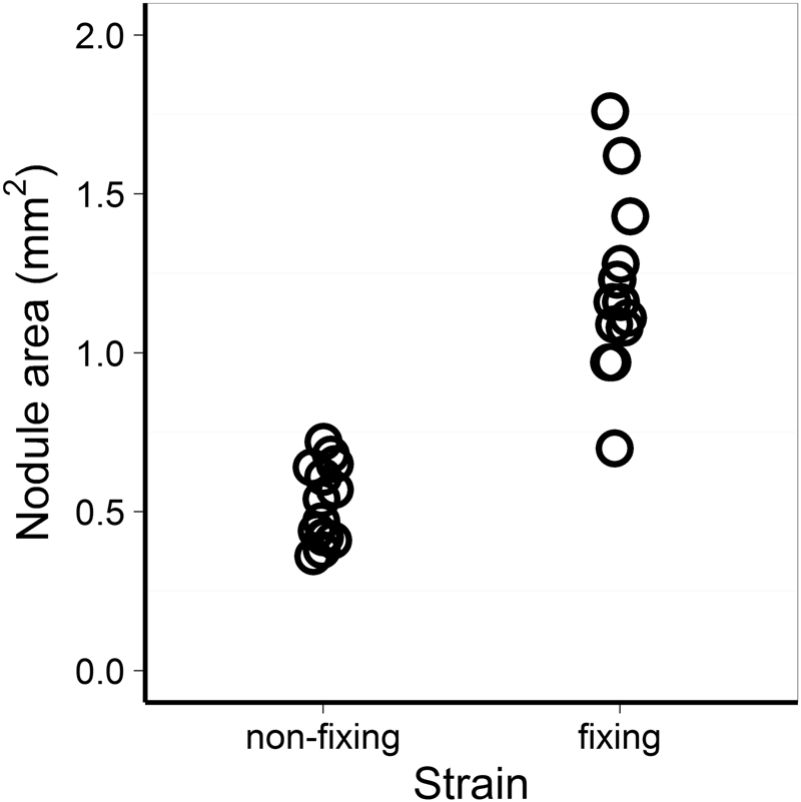
Legumes sanction non-fixing nodules. Nodules containing the fixing strain were significantly larger than nodules containing the fixing strain (paired t-test, *t*_*13*_ = 7.7176, *p* = 3.307 × 10^−6^), indicating that pea plants preferentially allocated resources to fixing nodules.

In the absence of partner choice, sanctions remain the only option for discriminating against non-fixing strains. If pea plants are able to sanction and differentially allocate resources on the basis of the nitrogen provided, we expect nodules containing a fixing strain to be larger than nodules containing a non-fixing strain. We therefore measured the sizes of a sample of nodules colonised by the different strains. Nodules containing the nitrogen-fixing strain were significantly larger than nodules containing the non-fixing strain (paired t-test, *t*_*13*_ = 7.7176, *p* = 3.307 × 10^−6^), indicating that pea plants preferentially allocated resources to fixing nodules, supporting sanctioning. Fixing nodules had an average area of 1.20 ± 0.07 mm^2^ (mean ± s.e.), and non-fixing nodules were 0.68 mm^2^ (95% CI [0.49; 0.87]) smaller – less than half of the area of fixing nodules (Fig. 3).

### Total number of nodules

**Figure 4:**
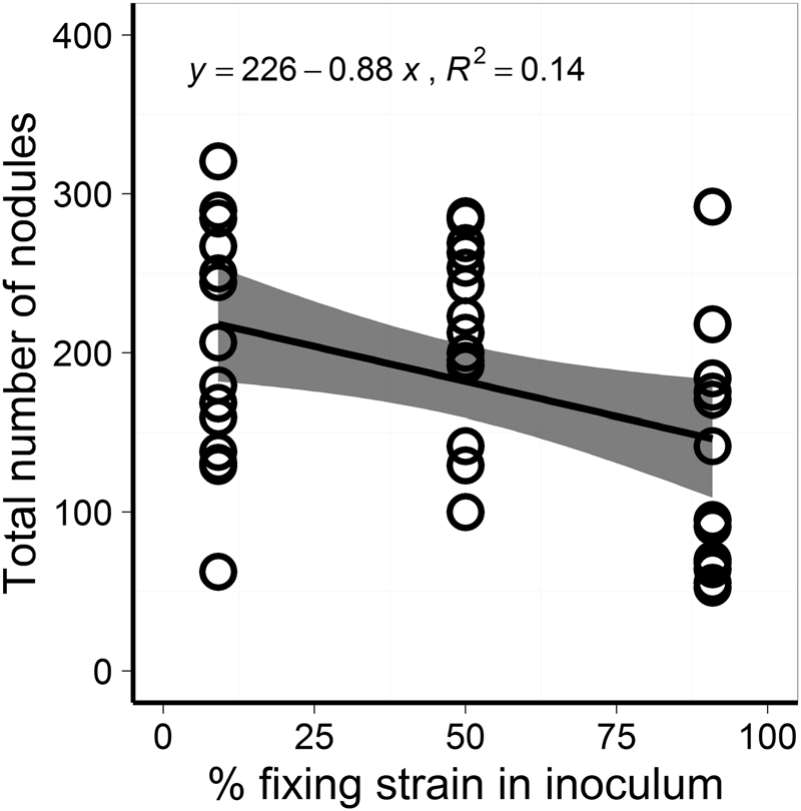
Total nodule number. The total number of nodules decreased as the percentage of fixing nodules increased (slope = −0.88 ± 0.34, *t*_*39*_ = −2.565, *p* = 0.0143). Regression with 95% confidence interval shown.

Each pea plant formed 183 ± 12 nodules (mean ± s.e.) on average. The total number of nodules (Fig. 4) decreased as the percentage of nitrogen-fixing nodules increased (slope = −0.88 ± 0.34, *t*_*39*_ = −2.565, *p* = 0.0143), probably because the nodulation process is inhibited once fixing nodules have been successfully established. A ten-fold increase in the percentage of nitrogen-fixing nodules resulted in 72 fewer nodules in total (95% CI [9, 135]) – a decrease of about 30%.

### Shoot mass

After three weeks of growth we found no differences in shoot dry mass among treatments, including water controls, (*F*_*3, 44*_ = 2.1921, *p* = 0.1024), presumably because the pea seeds contain large reserves of nitrogen. However, after five weeks of growth, independently grown plants inoculated with non-fixing RU3940 weighed 0.32 ± 0.04 grams (mean ± s.e.) while plants inoculated with the fixing strain Rlv3841 weighed 0.68 ± 0.04 grams, a significant difference of 0.37 grams (95% CI [0.23, 0.51]).

### Frequency of mixed nodules

**Figure 5:**
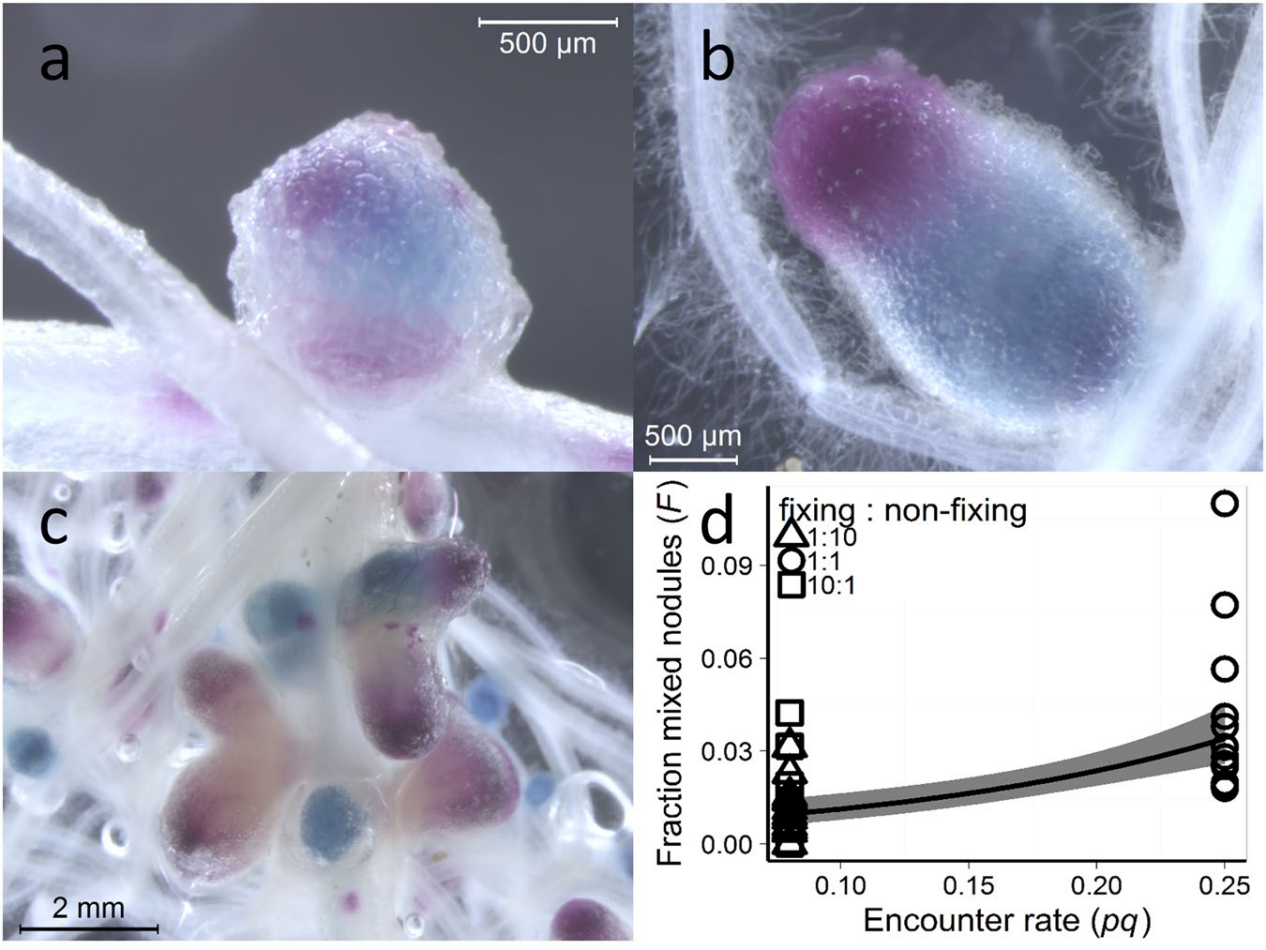
Mixed nodules. (**a)** Microscopic image of a mixed nodule on a pea root, which seems to have been infected by more than two rhizobia. The nitrogen-fixing strain is *celB* (blue) marked (3841 *celB*), and the non-fixing mutant strain is *gusA* (magenta) marked (OPS0365). (**b**) Microscopic image of a mixed nodule on a pea root infected by two rhizobia. The *gusA* marked nitrogen-fixing strain (3841 *gusA*) is magenta, and the *celB* marked non-fixing mutant strain (OPS0366) is blue. (**c**) Nodules, including mixed nodules, on a pea root. The *gusA* marked nitrogen-fixing strain (3841 *gusA*) is magenta, and the *celB* marked non-fixing mutant strain (OPS0366) is blue. (**d**) Mixed nodules occur more often when the probability that different strains encounter each other during nodule formation is higher (*t*_*39*_ = 4.913, *p* = 1.65 × 10^−5^). Generalized linear model with quasibinomial distribution and logit link function shown with 95% confidence interval.

The staining technique allowed clear visualization of mixed nodules, which occasionally appeared to result from multiple independent infections (Fig. 5A), but normally consisted of two – a fixing and a non-fixing strain (Fig. 5B,C). Mixed nodules occurred at an average frequency of 2.02% ± 0.35% (mean ± s.e.), and the frequency depended on the inoculum ratio (Fig. 5D). According to a simple diffusion model (in which we assume that the two rhizobial strains in the soil are well mixed) the predicted frequency of mixed nodules (*F*) is simply given by *F* = *pqε*, where *p* is the frequency of the fixing strain, *q* is the frequency of the non-fixing strain and *ε* is the unknown probability with which two bacteria simultaneously enter the same nodule; hence: *F ∝ pq*. We regressed *F* against *pq* (‘encounter rate’, Fig. 5D) and found that the frequency of mixed nodules indeed increased in proportion to the increase in encounter rate: a ± three-fold increase in encounter rate increased the odds of a mixed infection 3.5 times (95% CI [2.2, 5.9], *n* = 41). In an additional experiment, we varied the total inoculation density, as the diffusion model also predicts that mixed nodules occur more often at higher densities of rhizobia. As predicted, we found that the percentage of mixed nodules increased with inoculation density (slope = 1.87 ± 0.41, *t*_*18*_ = 4.545, *p* = 0.000251). A 10-fold increase in inoculation density, increased the percentage of mixed nodules by almost 2%.

### Fitness of rhizobial strains within mixed nodules

To assess the relative fitness of fixing versus non-fixing strains within mixed nodules, we measured the area occupied by each strain as indicated by the area stained blue versus magenta (or vice versa). We found that the percentage of nodule area occupied by the fixing strain ranged from 1.1 to 99%. Unfortunately, although the staining technique is valuable in visualizing mixed nodules, we conclude that it cannot be reliably used to assess the relative fitness of different strains within mixed nodules. The percentage of the nodule occupied by the fixing strain depended highly on the marker used to identify the fixing strain (*t*_218_ = 14.49, *p* < 2 × 10^−16^). If the fixing strain was marked with *gusA*, it appeared to occupy 31% of the nodule (95% CI [27.5; 33.6%]), but if the fixing strain was marked with *celB*, it appeared to occupy 75% of the nodule (95% CI [68.7; 80.0%].

## Discussion

We created a non-fixing but otherwise isogenic mutant to mimic a process that might occur in nature, where a mutation arises in a fixing rhizobial strain, rendering it ineffective. We found that pea plants could not discriminate between these fixing and non-fixing strains prior to nodule formation. Pea plants were therefore unable to detect whether the potential partner was effective at fixing nitrogen and could not prevent the formation of non-fixing nodules. Our results are supported by an early study using similar isogenic strains^27^, but this study was severely limited in sample size and has therefore been overlooked.

Our results indicate that partner choice is not a robust mechanism against ineffective strains as pea plants were unable to prevent non-fixing strains from entering. It could be argued that legumes may use genes other than *nifH* to assess the nitrogen fixation capacity of fixing strains before nodule formation; however we believe that this is unlikely. A mutation rendering a strain less effective can arise in any gene and effective partner choice would then require a mutation in the plant genome to detect this change. If the new mutation stops the cheat from entering, then it will spread through the plant population; however, given that rhizobial generation times are much faster than host plant generation times^28^, it seems that the host plants will be locked in an evolutionary arms race that they are doomed to lose; hence partner choice seems to be an ineffective way to stabilise the mutualism in the long term^21^. Furthermore, partner choice is susceptible to dishonest signals^13^.

In contrast, we found that pea plants did discriminate against ineffective strains via sanctioning, which has been previously reported using argon gas to replace atmospheric nitrogen^19–21^. In our experiment, nodules containing the non-fixing strain were roughly half the size of fixing nodules, indicating reduced plant resources. In contrast to the case of partner choice, sanctioning can stabilise the mutualism in the long-term. If a mutation arises that allows a plant to detect and sanction a partner that is not delivering the goods it would be effective against a wide variety of future ineffective strains. Thus, sanctioning allows an instantaneous response to ineffective strains and does not require specific recognition genes or rely on honest signals. Sanctioning is therefore a more robust^13^ mechanism against ineffective strains and can provide long-term stability to legume-*Rhizobium* mutualisms^16, 29^.

However, any discussion of sanctioning should take into account both plant and rhizobial fitness. Sanctioning can only be selected for when it saves plant resources and thus increases plant fitness. The reduced nodule size that we and others^20, 21^ have seen indicates that plants allocate fewer resources to non-fixing nodules. Whether this reduced resource allocation also reduces rhizobial fitness and thus stabilizes the mutualism on evolutionary time-scales is more difficult to establish. This may depend on whether or not the nitrogen-fixing bacteroids can still reproduce (usually determinate nodules), or are terminally differentiated and unable to reproduce (indeterminate nodules)^30^. In studies using argon gas, reduced rhizobial fitness has been shown in both determinate^19, 20^ and indeterminate^21^ non-fixing nodules. However, in a study using a non-fixing isogenic strain, the fitness of non-fixing rhizobia was not reduced in determinate soybean nodules up to five weeks old^31^. Because effects on rhizobial viability may emerge later in the sanctioning process, perhaps the best test would be a multigenerational experiment, where in the first generation plants are inoculated with both fixing and non-fixing strains, and new plants are then repeatedly grown in the same soil for several generations to see how quickly the non-fixing strain is eliminated. It would be of special interest to perform such an experiment with both indeterminate and determinate species.

Evidence from other mutualisms suggests that whether partner choice evolves in preference to sanctioning critically depends on how well partners can assess quality prior to establishment of the mutualistic relationship including the potential for dishonest signalling. For example, clients of the cleaner fish *Labroides dimidiatus* have evolved partner choice to counter cheating by individuals that take healthy tissue while removing parasites^32^. Partner choice is highly effective in this mutualism because the quality of service is known from previous experience and there are repeated interactions between individuals. In contrast, sanctioning has evolved in mutualisms between yucca moths and fig wasps and their respective plant hosts. In both cases the insects deposit seed-eating larvae in the flowers of host plants in return for pollination. In these mutualisms, plants cannot prevent eggs being laid nor assess partner quality, hence sanctioning has evolved: flowers containing too many eggs^33^, or too little pollen^33, 34^ are selectively aborted.

While our study shows that partner choice is not a robust mechanism to exclude ineffective strains, legumes do not form symbioses with all potential rhizobial strains. Instead, an extensive signalling process^35–37^ between legumes and their rhizobial partners can impose a high degree of selectivity on the relationship^36, 38, 39^, although the degree of selectivity varies greatly among hosts^39^. There are two explanations for this selectivity that are commonly proposed and are not mutually exclusive. First, specificity may arise in order to prevent the entry of pathogenic bacteria which utilise similar signalling pathways to gain access to host roots^38, 40, 41^. Second, by fine-tuning signalling pathways to target rhizobia that are particularly effective for a specific host, legume species might achieve greater nitrogen-fixation efficiency^42, 43^. This is likely to be true if host environments are sufficiently different that specialization by rhizobia is selected for. Support for specialization comes from the observation that a single rhizobial strain can vary greatly in its effectiveness among hosts^42^. This type of co-evolutionary process is separate from the need to avoid non-fixing rhizobia, which can arise by mutation at any time, in any strain, even those that are usually highly effective. That these two processes are indeed separate is supported by the fact that the genes involved in nitrogen fixation (*nif* and *fix* genes) are only expressed once the symbiosis has been established^44^ and are different from the signalling genes involved in infection (*nod* genes)^45^. Currently, genomic analyses are shedding more light on the selective pressures affecting both legume and rhizobial genes^46–48^.

Sanctioning is a robust mechanism against ineffective strains, but requires hosts to monitor partner quality and provide resources accordingly. Currently, little is known about the exact mechanism behind sanctioning in legume-*Rhizobium* symbioses, and whether it only takes place at the nodule level, or also occurs within nodules^13, 16^. If sanctioning takes place at the nodule level, mixed nodules could be a way for ineffective strains to avoid sanctions^6, 13, 49^. Indeed certain endosymbionts, even those belonging to different genera and lacking any genes for nitrogen fixation, have been shown to co-infect nodules by “piggybacking” on the genuine symbionts as they infect the root hairs^50^. Whether mixed nodules allow ineffective strains to persist depends on the frequency of mixed nodules, and on the relative fitness of fixing and non-fixing strains within mixed nodules. Estimates of the frequency of mixed nodules in the literature range from 2% to 74%^6, 51^. Our findings at least partly explain this variability as the frequencies we found could be adequately represented by a simple diffusion model, which predicts that more mixed nodules are expected: (1) at high rhizobial densities; (2) when the proportions of different strains are similar; and (3) when rhizobia diffuse more easily, which might occur, for example, under wet conditions. Although the staining technique is valuable in identifying strains and characterising mixed nodules, it could not be used reliably to assess fitness of rhizobial strains within mixed nodules. Further work on the mechanism of sanctioning, how it is affected by external conditions such as soil nitrogen, and how it affects rhizobial fitness will help illuminate how the legume-*Rhizobium* mutualism has persisted for much longer than humans have been around to reap its benefits.

## Methods

### Bacterial strains and culture conditions

We used *Rhizobium leguminosarum* bv. viciae (*Rlv*) 3841 strains labelled at identical positions in the chromosome with either a *gusA* or a *celB* marker gene^52^ (Table 1). Non-fixing mutants of these strains were made by replacing the *nifH* gene with a *nifH* gene disrupted by a spectinomycin resistance cassette (*nifH::ΩSpc*). *Rhizobium leguminosarum* only has a single copy of the *nifH* gene^53^, which is essential for nitrogen fixation^54^. First, an unmarked non-fixing strain was made, then the non-functional *nifH* gene was transduced into the marked fixing strains. To make the unmarked non-fixing strain, the *nifH* gene was amplified by PCR (2,660 bp product) using primers p950 and p951 that contain a SacI and SpeI restriction site and cloned into pCR2.1 in Dam- *E. coli* SCS110 (Stratagene) (pRU1907). A *Ω* spectinomycin cassette from pHP45Ω-Sp was cloned between two NruI sites within *nifH* resulting in a 166 bp deletion, and the *nifH::ΩSpc* fragment was cloned into the suicide vector pJQ200SK (pRU1908). Plasmid pRU1908 was conjugated into Rlv3841, and cells were plated on TY agar with gentamicin (20 μg ml^−1^) and then on AMS agar supplemented with 10% sucrose, 10 mM NH_4_Cl, and spectinomycin (100 μg ml^−1^) to select for gene replacement. Strain RU3940 was found to contain the correct *nifH::ΩSpc* mutation by PCR mapping using pOT forward with either p1002 or p1118 in separate reactions. To confirm differences in the nitrogen-fixing capacity of the two strains, we performed acetylene reductions on plants inoculated with either RU3940 or Rlv3841. RU3940 does not fix nitrogen (0.05 ± 0.02 (mean ± s.e, *n* = *3*) μmol ethylene per plant per hour, which is not different from water control plants (*t*_*6*_ = −0.025, *p* = 0.98)), whereas the parent strain Rlv3841 does fix nitrogen (2.58 ± 0.33 (mean ± s.e, *n* = *3*) μmol ethylene per plant per hour).

**Table 1:** Strains, plasmids and primers.

Strain, plasmid, or primer	Genotype or sequence	Reference of source
Strains		
Rlv3841	St^r^ derivative of *R. leguminosarum* bv. *viciae* strain 300	61
RU3940	Rlv3841 *nifH::Ω* Sp^r^	This work
3841 *gusA*	Rlv3841 with *gusA* marker	52
3841 *celB*	Rlv3841 with *celB* marker	52
OPS0365	3841 *gusA nifH::Ω* Sp^r^	This work
OPS0366	3841 *celB nifH::Ω* Sp^r^	This work
Plasmids		
pRU1907	*nifH* cloned in pCR2.1	This work
pHP45Ω-Sp	Plasmid containing Ω spec cassette, Sp^r^	62
pJQ200SK	pACYC derivative, P15A origin of replication, Gm^r^	63
pRU1908	*nifH::Ω*, Sp^r^ in pJQ200SK	This work
Primers		
pOTforward	CGGTTTACAAGCATAAAGC	
p1002	TTCCTCCATGTGCCTGGAGA	
p1118	GGTTCTTCGGAGTTTCTAT	
oxp0460	GCTTGATCATCGCCGGAAAC	
oxp0461	TGTCACCGCCGAAAACGATG	

The *nifH*::*ΩSpc* cassette from non-fixing strain RU3940 was transduced into the *gusA* or *celB* marked strains using phage RL38^55^, yielding non-fixing *gusA* (OPS0365) and *celB* (OPS0366) marked strains which are otherwise isogenic to their fixing *gusA* and *celB* marked parent strains. Correct insertion of the *nifH::ΩSpc* cassette from RU3940 was confirmed by PCR mapping using pOT forward with either oxp460 or opx461 in separate reactions (Supplementary Fig. S1). Enzyme assays on free living cultures confirmed conservation of the *gusA* and *celB* marker genes in the non-fixing mutants (Supplementary Fig. S2 and Supplementary Fig. S3 respectively). Bacterial cultures were maintained on TY agar^56^ with 500 μg ml^−1^ streptomycin (all strains) and 100 μg ml^−1^ spectinomycin (RU3940, OPS0365 and OPS0366 only).

### Plant growth

Pea (*Pisum sativum* cv Avola) seeds were germinated in the dark for five days on agar plates at room temperature and then transferred to 500 ml pots containing a mixture of silver sand - fine vermiculite (1:1 v/v) substrate, 75 ml nitrogen free nutrient solution (as in Poole *et al*.^57^ but 2.67 times more concentrated) and 1 ml rhizobial inoculum. Peas were grown in the growth room at 21 °C with a 16 hour photoperiod.

### Experimental designs

To test for partner choice, we applied rhizobial inocula consisting of 1:10, 1:1 and 10:1 ratios of fixing to non-fixing strains. To exclude any effect of the *gusA* or *celB* marker genes, both combinations were tested: fixing *gusA* (3841 *gusA*) with non-fixing *celB* (OPS0366) strains and fixing *celB* (3841 *celB*) with non-fixing *gusA* (OPS0365) strains. Total inoculum density for all treatments was 1 × 10^4^ cells per pot. Rhizobial cultures for the inocula were spread-plated to confirm numbers of rhizobia in the inocula. In a fully randomized design, we grew 49 plants in total: six treatments plus a water control (no rhizobia), all replicated seven times. Plants were grown for 21 days without additional watering.

To assess the effect of inoculation density on the frequency of mixed nodules, plants were inoculated with 1:1 ratios of fixing *gusA* (3841 *gusA*) to non-fixing *celB* (OPS0366) strains at total densities of 1 × 10^2^, 1 × 10^3^, 1 × 10^6^ and 1 × 10^8^ cells per pot. In a fully randomized design, we grew five plants at each inoculum density plus two water controls. Plants were grown for 38 days and watered with sterilized water as needed after 21 days.

To test for differences in markers on the percentage of the nodule area occupied by the fixing strain, six plants were grown in an additional independent experiment, inoculated with 1:1 ratios of fixing *celB* (3841 *celB*) to non-fixing *gusA* strains (OPS0365) at a total density of 1 × 10^8 cells per pot. Plants were grown for 37 days and watered with sterilized water as needs after 21 days.

To assess the phenotype of non-fixing mutant strain RU3940, plants were inoculated with either fixing strain Rlv3841 or non-fixing strain RU3940 and grown for four or five weeks in independent experiments.

### Harvest

At harvest, roots were gently washed and then stained for *gusA* and *celB* marker genes^58^. In aluminium covered tubes, roots were incubated overnight at 28 °C submerged in phosphate buffer (7 g L^−1^ NaH_2_PO_4_, 7.2 g L^−1^ Na_2_HPO_4_, 1 mM EDTA (pH 8), 1% Sarkosyl, 1 ml L^−1^ Triton) supplemented with 0.2 mg ml^−1^ Magenta-glc (5-bromo-6-chloro-3-indolyl-β-D-glucuronide). In fresh phosphate buffer, roots were then incubated at 70 °C for 1 hour 45 min. After cooling down, X-gal (5-bromo-4-chloro-3-indolyl-β-D-galactopyranoside) was added to a final concentration of 0.25 mg ml^−1^ and roots were incubated overnight at 37 °C. This yielded visibly magenta (*gusA* marked strains), blue (*celB* marked strains) or magenta and blue (mixed) nodules (Fig. 2A–C). Shoots were dried at 70 degrees Celsius for 24–72 hours and then weighed.

### Data collection

Roots were carefully laid out so that all nodules were visible and then photographed. Two people independently counted blue and magenta nodules from photographs taken of whole roots, zooming in as necessary, and the average was taken as the final count. Mixed nodules were counted and photographed using a dissecting microscope (Leica M165 FC) with accompanying software (LAS v4.5). To estimate size of nodules containing fixing versus non-fixing strains, we measured the area of nodules from the photographs taken of the whole root, using ImageJ v1.49v^59^, which allowed for measuring areas of irregular shapes. Ten fixing and ten non-fixing nodules were randomly selected for each of the 14 plants inoculated with a 1:1 ratio of fixing to non-fixing strains (7 of which had the *gusA* marked fixing strain and 7 had the *celB* marked fixing strain). To estimate the percentage of mixed nodules that was occupied by the fixing strain, we measured the areas of blue and magenta in all mixed nodules for which this was possible (distinct enough areas of blue and magenta), using ImageJ. Additional microscopy was done on whole nodules (five-week-old nodules). Images were taken with a dissecting microscope (Leica M165 FC) with accompanying software (LAS v4.5).

### Statistical analyses

We tested for partner choice by regressing the percentage of nodules containing nitrogen-fixing rhizobia against the percentage of nitrogen-fixing rhizobia in the inoculum. If plants do not exert partner choice, we expect a 1:1 relationship (a slope of 1.0). Water controls and mixed nodules were excluded from this analysis. To test whether the presence of *gusA* versus *celB* marker genes affected competitive ability, we carried out an ANCOVA with the marker gene as the categorical variable. Results presented are from the regression model without this interaction, as marker genes did not affect the slope (*t*_37_ = −0.189, *p* = 0.851) or intercept (*t*_37_ = −0.177, *p* = 0.861) of the regression line. The intended inoculation ratios were used as the explanatory variable, as it was confirmed using colony counts that the actual ratios in inocula did not deviate by more than 11% from intended inoculation ratios (*n* = 6, mean ± s.e. 3.78 ± 1.49%, Supplementary Table S1). Accounting for this deviation did not change results, and if anything would lead to a decrease of the estimate of the slope (whereas for partner choice we would expect an increase of the estimate of the slope).

To test for differences in size between fixing and non-fixing nodules, we performed a paired t-test on the average size of ten nodules, pairing fixing and non-fixing nodules of each plant. To test whether the total number of nodules formed depended on the ratio of fixing to non-fixing strains in the inoculum, we regressed the total number of nodules against the percentage of fixing strain in the inoculum. Shoot mass was also regressed against the percentage of the fixing strain in the inoculum. To assess the frequency of mixed nodules, we analysed how the occurrence of mixed nodules depended on the inoculation ratio of fixing to non-fixing strains with a generalized linear model using a quasibinomial distribution and a logit link function (because data were expressed as proportions and there was significant overdispersion). We also regressed the percentage of mixed nodules against the log of the total inoculation density. Whether the fixing strain was marked with *gusA* or *celB* had no impact on the proportion of mixed nodules detected (*t*_*37*_ = 0.494, *p* = 0.62493) or on the interaction with the encounter rate (*t*_*37*_ = 1.137, *p* = 0.26301), so we present results from analyses which do not include this interaction. To assess fitness of fixing to non-fixing strains within mixed nodules, we used a linear model testing for the effect of the marker (*gusA* or *celB*) used to mark the fixing strain. Individual nodules were seen as the unit of replication, and this was confirmed to be a valid assumption as the plant from which nodules came accounted for only 9% of the variation in the data. All analyses were performed in R version 3.0.2^60^.

## Electronic supplementary material

Supplementary Information
