# Supplementary material for: Policing the legume-Rhizobium symbiosis: a critical test of partner choice
Source: Sci Rep. 2017 May 3;7:1419. doi: 10.1038/s41598-017-01634-2 (PMC5431162; doi:10.1038/s41598-017-01634-2)
Supplement: Supplementary file 1 — Supplementary Information [file 41598_2017_1634_MOESM1_ESM.pdf]

Supplementary information

Policing the legume-Rhizobium symbiosis: a critical test of partner choice.

Annet Westhoek[1, 2], Elsa Field[1], Finn Rehling[1,3], Geraldine Mulley[4], Isabel Webb[1], Philip S. Poole[1*] & Lindsay A. Turnbull[1*]

[1]Department of Plant Sciences, University of Oxford, Oxford OX1 3RB, UK. [2]Systems Biology Doctoral Training Centre, University of Oxford, Oxford OX1 3RQ, UK. [3]Department of Ecology, Philipps-University Marburg, Marburg D-35043, Germany. [4]School of Biological Sciences, University of Reading, RG6 6AJ, UK. Correspondence should be sent to PSP (philip.poole@plants.ox.ac.uk) and/or LAT (lindsay.turnbull@plants.ox.ac.uk).

Supplementary figure 1

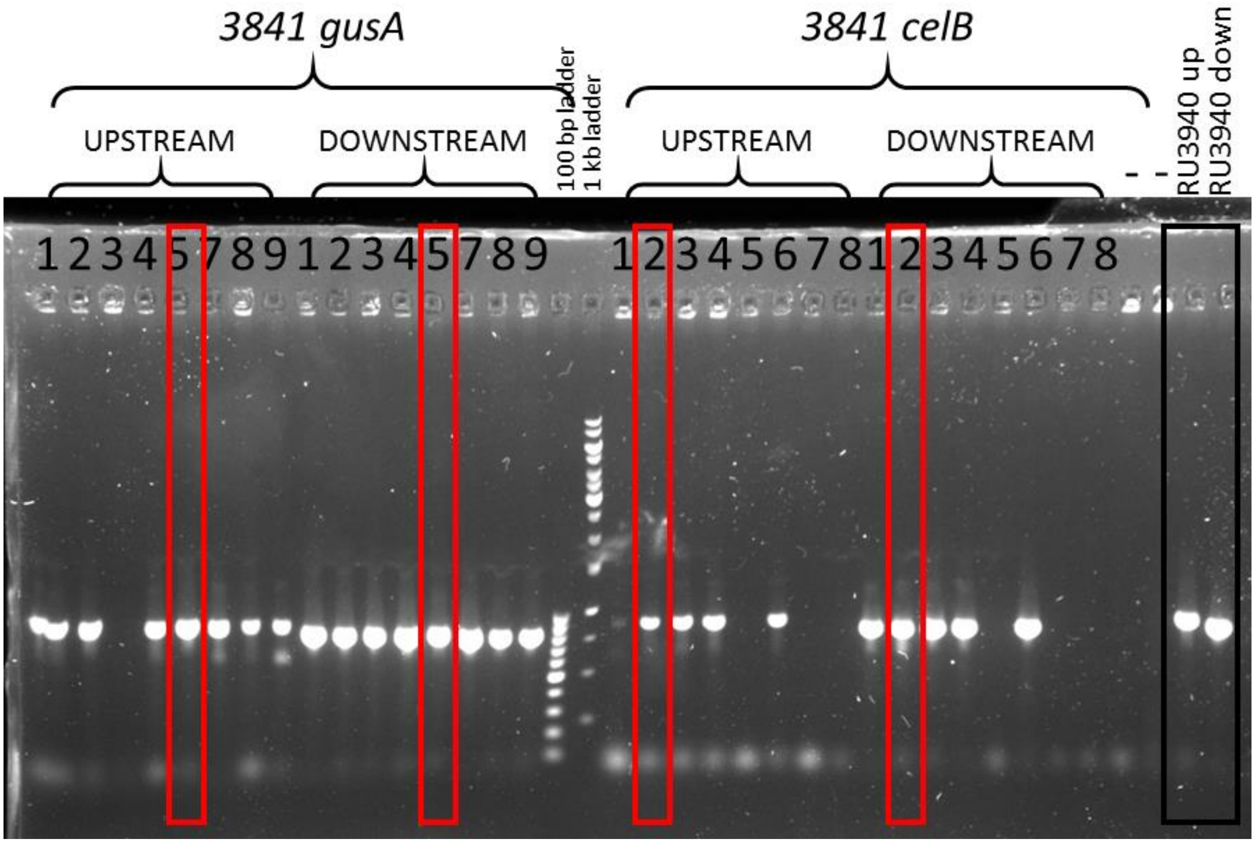

Supplementary Figure 1. Confirmation of successful insertion of *nifh::Ωspec* mutation into *gusA* and *celB* marked strains. Successful insertion yields *gusA* and *celB* marked *nifh::Ωspec* (non-fixing) mutants. Primers were designed to bind in the *Ωspec* cassette and upstream and downstream of the *nifH* gene, yielding fragments upstream and downstream of the *Ωspec* cassette respectively. If the insertion worked correctly, PCR fragments upstream and downstream of the insertion have the same length in the marked mutants as in the original unmarked *nifh::Ωspec* mutant (RU3940, black box). Multiple strains were made (8 *celB* strains and 9 *gusA* strains (strain 6 not included in PCR because *gusA* marker could not be confirmed)), some of which could not be confirmed. *gusA* marked strain 5 (OPS0365) and *celB* marked strain 2 (OPS0366) were used in this study (red boxes).
Primers used for upstream fragment: oxp0460 (GCTTGATCATCGCCGGAAAC), binding before the start codon of the *nifH* gene, and pOT forward (CGGTTTACAAGCATAAAGC), binding in the *Ωspec* cassette. Primers used for downstream fragment: pOT forward (binds in two places in *Ωspec* cassette) and oxp0461 (TGTCACCGCCGAAAACGATG), binding downstream of the *nifH* gene.

Supplementary figure 2

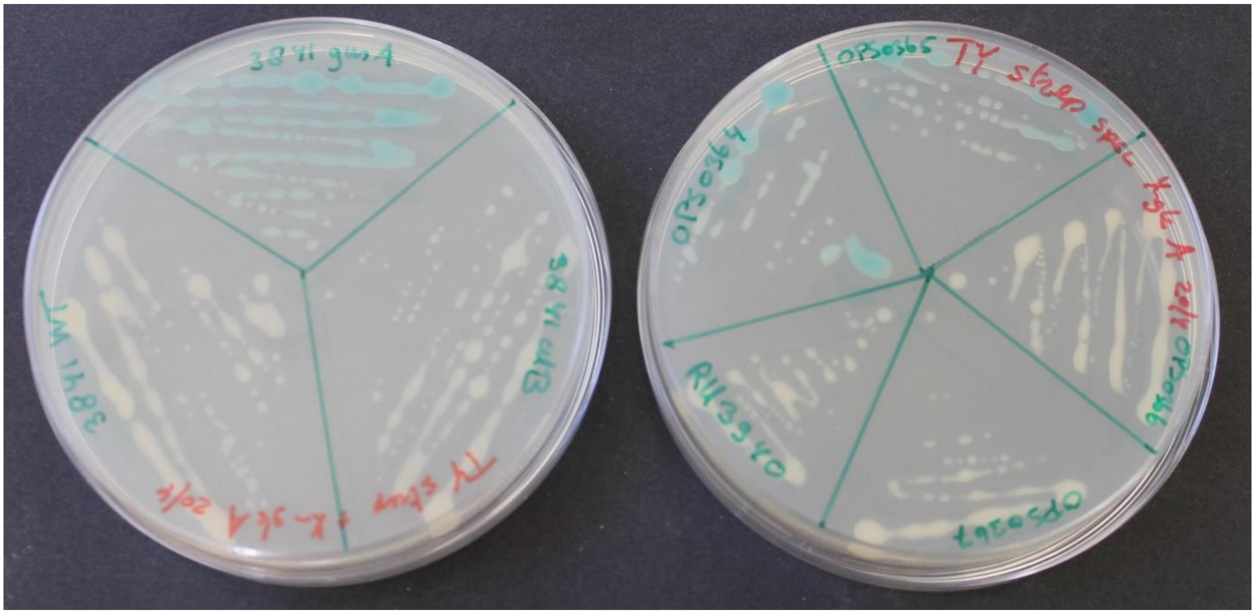

Supplementary Figure 2. Confirmation conservation of *gusA* marker gene. It was confirmed that the novel strains with the *nifh::Ωspec* insertion still have the original *gusA* marker. The left hand plate contains the nitrogen-fixing strains on TY medium with streptomycin and X-glcA, the substrate which yields a blue colour if there is *gusA* encoded β-glucuronidase activity. Only the *gusA* marked strain is blue (top section), not the unmarked wild type and *celB* marked strain, as expected. The plate on the right hand side contains *nifh::Ωspec* mutants on TY medium with streptomycin, spectinomycin and X-glcA. Again, only the *gusA* marked strains OPS0364 (not used in this study) and OPS0365 (used in this study) are blue (top two sections).

Supplementary figure 3

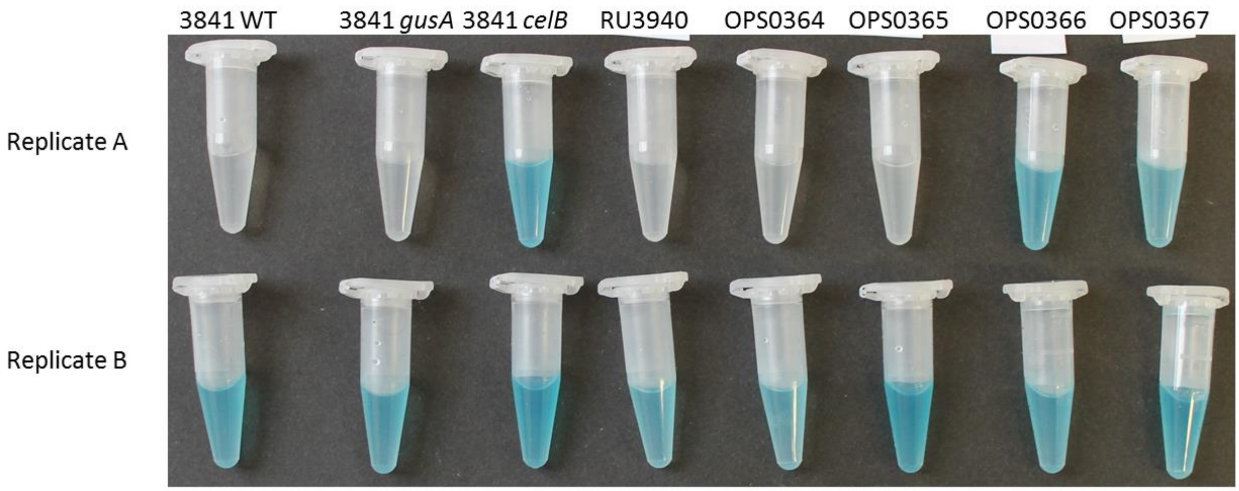

Supplementary Figure 3. Confirmation conservation of *celB* marker gene. It was confirmed that the novel strains with the *nifh::Ωspec* insertion still have the original *celB* marker. Free living cultures provided with X-gal yield a blue colour if there is *celB* encoded β-galactosidase activity. Replicate B (bottom) was not heat treated, so the endogenous β-galactosidase activity yields a blue colour for all strains. Replicate A (top) was heat treated, during which endogenous β-galactosidase was destroyed. Only the thermostable *celB* marked strains (*celB* marked nitrogen-fixing 3841, and OPS0366 (this study) and OPS0367) yield a blue colour. Unmarked original wild type 3841 and *nifh::Ωspec* (RU3940) are white, just as *gusA* marked strains OPS0364 and OPS0365.

Supplementary table 1

Ratio fixing:non-fixing strain	Fixing strain	Intended percentage fixing strain	Estimated achieved percentage fixing strain	Deviation (percentage)
1:10	*gusA*	9.09	13.5	4.5
1:10	*celB*	9.09	10.0	1.0
1:1	*gusA*	50	60.7	10.7
1:1	*celB*	50	52.7	2.7
10:1	*gusA*	90.9	93.9	3.0
10:1	*celB*	90.9	91.8	0.9

Table 1. Intended and achieved percentage of fixing strain in the inocula.
